# 3-Hydroxytyrosol as a phenolic cholinesterase inhibitor with antiamnesic activity: a multimethodological study of selected plant phenolics

**DOI:** 10.3389/fphar.2025.1640034

**Published:** 2025-10-07

**Authors:** Tugba Ucar Akyurek, F. Sezer Senol Deniz, Ipek Suntar, Gokcen Eren, Onur Kenan Ulutas, Ilkay Erdogan Orhan

**Affiliations:** ^1^ Department of Pharmacognosy, Faculty of Pharmacy, Gazi University, Ankara, Türkiye; ^2^ Department of Pharmaceutical Chemistry, Faculty of Pharmacy, Gazi University, Ankara, Türkiye; ^3^ Department of Pharmaceutical Toxicology, Faculty of Pharmacy, Gazi University, Ankara, Türkiye; ^4^ Department of Pharmacognosy, Faculty of Pharmacy, Lokman Hekim University, Ankara, Türkiye

**Keywords:** alzheimer’s disease, antiamnesic activity, cholinesterase inhibition, molecular docking, phenolic compounds

## Abstract

**Background:**

Plant phenolics are increasingly being investigated for their diverse biological activities, including neuroprotective effects relevant to conditions like Alzheimer’s disease.

**Objective:**

The neurobiological potential of 37 plant phenolics was screened through a multifaceted approach encompassing *in vitro* enzyme inhibition and antioxidant assays, *in vivo* antiamnesic evaluation, and *in silico* molecular docking and toxicity predictions.

**Methods:**

The compounds were tested for their cholinesterase (ChE) inhibition potentials, metal-chelation activities, and copper-reducing antioxidant capacities (CUPRACs) using a microtiter assay as well as ferric-reducing antioxidant power assays. Additionally, the *in silico* ADME, pharmacokinetic, and toxicokinetic profiles of the compounds were predicted using computational platforms.

**Results:**

Several compounds exhibited significant inhibition of acetylcholinesterase (AChE) and butyrylcholinesterase (BChE) activities. Of these, quercetin was found to be the most active inhibitor, with IC_50_ values of 1.22 ± 0.79 mM against AChE and 2.51 ± 0.04 mM against BChE. Some of the other compounds, including caffeic acid (IC_50_: 3.51 ± 0.62 mM), apigenin (IC_50_: 3.52 ± 0.08 mM), and taxifolin (IC_50_: 7.18 ± 2.05 mM), also showed AChE inhibition. Then, oleuropein, rosmarinic acid, gallic acid, epigallocatechin gallate, and 3-hydroxytyrosol were further investigated for their antiamnesic activities using a passive avoidance test in scopolamine-induced mice; our data showed that these compounds were effective considering the latency time of the mice and that 3-hydroxytyrosol showed the highest antiamnesic effect. The dual inhibitory compounds were subjected to molecular docking experiments with ChEs, and the *in silico* toxicities of three compounds were assessed using the PASS and SwissADME prediction programs.

**Conclusion:**

Our data provide compelling evidence for the neuroprotective potentials of several plant phenolics. Notably, 3-hydroxytyrosol was identified for the first time as a ChE inhibitor with significant *in vivo* antiamnesic activity and warrants further investigation.

## Introduction

Natural products are increasingly being considered inspiring lead molecules in pharmaceutical research. In this sense, numerous molecules have been identified from herbal sources that have shown remarkable neurobiological effects (Wasik and Antkiewicz-Michaluk, 2017; Khadka et al., 2020; Zhu et al., 2022). Neurodegeneration is broadly defined as mass gradual neuron loss and atrophy and comprises many conditions like Alzheimer’s disease (AD), Parkinson’s disease, Huntington’s disease, multiple sclerosis, and amyotrophic lateral sclerosis, all of which progress via complex pathological mechanisms (Dugger and Dickson, 2017). Among these, AD is the commonest type of dementia and is a progressive neurological disease with a high incidence rate, especially in the elderly population, which makes it burdensome in terms of pharmaceutical care (Niu et al., 2017). The AD type of dementia constitutes approximately 50%–60% of all dementia cases seen over the age of 65 today (Niu et al., 2017; Lopez and Kuller, 2019). The most prominent or characteristic symptom of AD is memory loss (Jahn, 2013; Kirova et al., 2015), for which there is no available cure to impede the disease but only symptomatic treatment at the moment. The cholinergic hypothesis is the most accepted theory to describe the pathology of AD and is related to a deficit in acetylcholine that is hydrolyzed by the enzyme acetylcholinesterase (AChE) (Anand and Singh, 2013; Saxena and Dubey, 2019). Another target enzyme in the cholinergic system is butyrylcholinesterase (BChE), which is reportedly linked to the pathology of AD via excess accumulation in amyloid beta (Aβ) plaques in the brains of AD patients (Darvesh, 2016; Xing et al., 2021). Although the amyloid hypothesis is another widely accepted theory associated with the pathology of AD, no relevant drugs have been developed till date that act through this mechanism (Gouras et al., 2015; Paroni et al., 2019).

The most prescribed class of drugs for the treatment of AD is cholinesterase (ChE) inhibitors (i.e., rivastigmine, tacrine, donepezil, and galantamine), along with *N*-methyl-D-aspartate (NMDA) receptor antagonists like memantine for pharmacotherapy of the disease (Dou et al., 2018; Guo et al., 2020). It is noted that galantamine, which is the latest generation of ChE inhibitory drugs, is originally a natural product that was initially isolated from the bulbs of *Galanthus woronowii* Losinsk (snowdrop plant, Amaryllidaceae); over time, it has been developed into a clinical drug for symptomatic therapy of AD (Heinrich and Lee, 2004; Naguy et al., 2022). Relevantly, physostigmine (syn. eserine) is another natural compound obtained from *Physostigma venenosum* Balf (locally known as Calabar bean, Fabaceae); it was earlier used as the first-line ChE inhibitor against AD and has since been considered as a chemical model or lead molecule for designing next-generation ChE inhibitors (Mohs et al., 1985; Pinho et al., 2013). Thus, we can foresee the utility of leading natural products for treating AD. Accumulation of metals like aluminum, iron, and copper in the Aβ plaques in the brains of AD patients has been frequently reported to contribute to its pathology; thus, metal chelation has been proposed to assist with AD therapy (Das et al., 2021; Singh et al., 2022). Many relevant studies have shown that inhibition of enzymes like ChE, secretase family, and prolyl endopeptidase can be considered first-step assays for determining the neuroprotective effects against AD since the currently available clinical drugs against AD are mostly ChE inhibitors. Neuroprotection can also be tested via several *in vivo* experimental models like the passive avoidance test, Y-maze test, and zebrafish assays.

The search for effective treatments to combat the debilitating conditions of AD remains a critical priority in pharmaceutical research. Many studies have reported that plant polyphenols, particularly flavonoids, have encouraging effects against AD and other neurodegenerative diseases through various mechanisms (Lopez and Kuller, 2019; Firdaus and Singh, 2021). Preclinical studies help elucidate the underlying mechanisms by which plant phenolics exert their neuroprotective effects. In the present work, previous outcomes based on pertinent plant phenolics inspired us to screen 37 selected phenolic compounds shown in Table 1 for possible neuroprotective effects; these compounds were mainly selected on the basis of their wide availability in medicinal plants and neuroprotective potential using a combination of *in vitro*, *in vivo*, and *in silico* methods. Given these two reasons, the selection of compounds was also based on the limited data of their exploitation for the activities studied, variations in the experimental methods used for identical activities, use of different enzyme sources, purity and stability, and differences in the dose administration or concentrations, which can be explained in most cases. In addition, bioactivity studies on natural compounds in their pure forms are rather limited compared to those on herbal extracts. Although previous *in silico* and *in vitro* studies have suggested the anticholinesterase potentials of several of these phenolic compounds, there remains a gap in literature regarding the direct biological effects. Specifically, there is a lack of evidence from a multifaceted approach combining *in vitro* enzyme assays with behavioral *in vivo* models to confirm the neuroprotective efficacies. Therefore, of the 37 plant phenolics considered herein, five compounds with dual ChE inhibition effects, namely, oleuropein, rosmarinic acid, gallic acid, epigallocatechin gallate (EGCG), and 3-hydroxytyrosol, in amounts sufficient for the *in vivo* experiments were subjected to a passive avoidance test in scopolamine-induced amnesic mice. In particular, we note that no prior studies have demonstrated the antiamnesic effects of 3-hydroxytyrosol in living organisms. The molecular interactions of these inhibitory compounds were examined through molecular docking simulations, while their possible *in silico* toxicity effects were assessed using the prediction of activity spectra for substances (PASS) and SwissADME programs.

**TABLE 1:** AChE and BChE inhibitory effects (inhibition% ± SD) as well as IC_50_ values of the tested phenolics.

No.	Phenolic compounds	Chemical class	Enzyme inhibition (% ± SD^a^) at 10 mM and IC_50_ values	
			AChE	BChE
1	Gallic acid	Hydroxybenzoic acid	79.23 ± 0.54 (IC_50_: 4.22 ± 0.07 mM)	84.40 ± 2.25 (IC_50_: 3.29 ± 0.32 mM)
2	Syringic acid		2.39 ± 4.18	17.56 ± 4.24
3	Vanillic acid		-^b^	35.06 ± 3.21
4	Chlorogenic acid	Ester of caffeic acid and (-)-quinic acid	18.67 ± 1.76	25.35 ± 1.72
5	Ellagic acid	Dimeric derivative of gallic acid	—	—
6	*Trans*-ferulic acid	Hydroxycinnamic acid	6.26 ± 2.55	14.91 ± 11.10
7	*p-*Coumaric acid		—	5.15 ± 3.96
8	Caffeic acid		57.33 ± 4.54 (IC_50_: 3.51 ± 0.62 mM)	47.82 ± 2.75
9	Rosmarinic acid	Caffeic acid ester	69.99 ± 1.84 (IC_50_: 3.08 ± 0.50 mM)	68.91 ± 4.51 (IC_50_: 6.14 ± 0.09 mM)
10	D-(-)-quinic acid	Cyclohexanecarboxylic acid	5.44 ± 4.01	5.84 ± 4.60
11	*p*-Tyrosol	Phenethyl alcohol	—	35.58 ± 3.54
12	3-Hydroxytyrosol		62.40 ± 0.68 (IC_50_: 4.56 ± 0.27 mM)	94.35 ± 2.40 (IC_50_: 6.96 ± 3.15 mM)
13	Oleuropein	Secoiridoid	84.10 ± 3.11 (IC_50_: 5.39 ± 0.35 mM)	90.54 ± 2.51 (IC_50_: 6.48 ± 1.03 mM)
14	Pelargonidin	Anthocyanidin	34.25 ± 0.50	25.29 ± 4.13
15	*Trans-*resveratrol	Stilbene	54.02 ± 3.59 (IC_50_: 5.19 ± 2.83 mM)	53.21 ± 5.13 (IC_50_: 9.87 ± 1.01 mM)
16	Catechin	Flavan-3-ol	—	—
17	Epigallocatechin gallate (EGCG)		69.11 ± 3.91 (IC_50_: 4.85 ± 2.03 mM)	84.43 ± 0.86 (IC_50_: 5.04 ± 0.29 mM)
18	Quercetin	Flavonol	80.98 ± 1.24 (IC_50_: 1.22 ± 0.79 mM)	99.40 ± 0.76 (IC_50_: 2.51 ± 0.04 mM)
19	Kaempferol		30.91 ± 1.83	—
20	Myricetin		—	—
21	Fisetin		96.21 ± 0.06 (IC_50_: 1.28 ± 1.28 mM)	76.19 ± 2.48 (IC_50_: 9.62 ± 0.81 mM)
22	Morin		9.72 ± 0.96	2.81 ± 3.83
23	Luteolin	Flavone	—	—
24	Apigenin		76.97 ± 1.50 (IC_50_: 3.52 ± 0.08 mM)	—
25	Taxifolin	Flavononol	50.43 ± 1.12 (IC_50_: 7.18 ± 2.05 mM)	14.36 ± 1.71
26	Tricin	Methoxyflavone	—	10.48 ± 4.13
27	Tangeretin		—	9.97 ± 4.09
28	Diosmetin		—	18.94 ± 2.07
29	Hesperetin	Flavanone	19.19 ± 2.75	25.58 ± 3.92
30	Naringin	Flavanone 7-O-glycoside	4.27 ± 4.28	14.94 ± 0.13
31	Isoquercitrin	Flavone 3-O-glycoside	43.17 ± 1.65	31.77 ± 0.39
32	Orientin	Flavone 8-C-glycoside	—	—
33	Vitexin	Flavone 8-C-glycoside	17.32 ± 2.77	23.14 ± 3.30
34	Daidzein	Isoflavone	46.63 ± 16.87	19.62 ± 7.10
35	Genistein		13.05 ± 0.5	—
36	Glycitein		—	29.77 ± 0.01
37	Amentoflavone	Biflavonoid	—	—
Reference	Galantamine	Alkaloid	98.34 ± 0.72 (IC_50_: 0.0018 ± 0.00005 mM)	87.22 ± 2.08 (IC_50_: 0.25 ± 0.01 mM)

^a^ Standard deviation (n = 3).

^b^ No inhibition.

## Materials and methods

### Chemicals

The reference phenolic compounds used in this study (Table 1) as well as galantamine (reference drug) and scopolamine hydrobromide (S1875-5G) were acquired from Sigma-Aldrich (St. Louis, MO, United States); all compounds had purity values over 95%.

### Microtiter assay for ChE inhibition

Inhibitory activities of electric eel AChE (Type VI-S, EC 3.1.1.7, Sigma, St. Louis, MO, United States) and horse serum BChE (EC 3.1.1.8, Sigma) were determined using Ellman’s method with slight modifications (Ellman et al., 1961). Briefly, 140 µL of 0.1 mM sodium phosphate buffer (pH 8), 20 µL of 5,5′-dithio-bis (2-nitrobenzoic) acid (DTNB, 0.4 mM, Sigma), 20 µL of the enzyme (either AChE or BChE, 5.32 × 10^−3^ U), and 20 µL of the test sample were added to the reaction mixture and incubated for 15 min at 25 °C. Next, approximately 10 µL of acetylthiocholine iodide (0.4 mM) or butyrylthiocholine chloride (0.4 mM) was added to the incubated sample as the substrate reacting with DTNB to form 5-thio-2-nitrobenzoate anion, which resulted in a yellow-colored complex. The absorbance was then measured at 417 nm using a 96-well ELISA microplate reader (VersaMax, Molecular Devices, San Jose, CA, United States). Here, galantamine hydrobromide was used as the reference drug.

### Data processing for enzyme inhibition assays

The measurements and calculations were processed using Softmax PRO 4.3.2. LS software. The percentage inhibitions of AChE and BChE were determined by comparing the reaction rates of the test samples with those of blank samples. The extent of the enzymatic reaction was calculated using the equation I% = [(C–T)/C] × 100, where I% is the activity of the enzyme given as percentage inhibition, C is the absorbance of the control solvent (blank) in the presence of the enzyme, and T is the absorbance of the test sample or positive control/reference inhibitor (i.e., galantamine) in the solvent in the presence of the enzyme. This value expresses the effect of the test sample or positive control/reference inhibitor on AChE or BChE enzymatic activity in terms of the percentage of remaining activity in the presence of the test sample or positive control. The data were expressed as average inhibition ± standard deviation (SD), and the results were taken from at least three independent experiments performed in triplicate.

### Passive avoidance test in mice

Male Swiss albino mice (20–25 g) were used in the experiments. The animal study was conducted in adherence to the Animal Research: Reporting of *In Vivo* Experiments guidelines, and all experimental procedures were approved by the Local Animal Ethics Committee of Gazi University (approval number: G.Ü.ET-19.057; 4 October 2019). The animals were housed under controlled laboratory conditions (temperature: 21–24 °C; 12-h light/12-h dark cycles) for at least 1 week prior to the start of the experiments. During testing, the background noise was minimized, and the experiment room was consistently illuminated according to standard protocols. Throughout the study, the animals were provided a standard pellet feed and water *ad libitum*. Following the 1-week acclimatization period, the mice were randomly divided into eight groups, with eight mice per group. This group size was based on previous studies employing similar behavioral models, where a minimum of eight animals per group was reported (Orhan and Aslan, 2009; Getova and Mihaylova, 2013; Barkur and Bairy, 2015; Budzynska et al., 2015). The eight groups were as follows: A: 0.9% NaCl (sham), B: scopolamine hydrobromide (control), C: oleuropein, D: 3-hydroxytyrosol, E: gallic acid, F: rosmarinic acid, G: EGCG (phenolic compound), and H: galantamine (reference). During the acquisition phase of the passive avoidance test, each mouse was first placed in a light compartment; the guillotine door was opened 10 s later, and the step-through latency of the animal to a dark compartment was recorded. Upon entry, the door to the compartment was closed automatically, and a foot shock of 0.1 mA/10 g of body weight was delivered for 3 s (Orhan and Aslan, 2009). Immediately after the acquisition phase, the animals received their respective treatments: group A (sham) received only 0.9% NaCl; groups C–G were administered oleuropein, 3-hydroxytyrosol, gallic acid, rosmarinic acid, and EGCG, respectively, where each compound was prepared in 0.9% NaCl at a concentration of 20 mg/kg and delivered via intraperitoneal (*i.p.*) injection; group H received the reference drug galantamine at a dose of 10 mg/kg via subcutaneous (*s.c.*) injection (De Bruin and Pouzet, 2006). Thirty minutes after treatment, groups B–H received scopolamine hydrobromide (1 mg/kg, dissolved in 0.9% NaCl) via *i.p.* injection to induce amnesia. Twenty-four hours after scopolamine administration, the mice were reintroduced to the light compartment, and their step-through latencies to the dark compartment were measured. Mice that did not enter the dark compartment within 9 min (cutoff time) were considered to have retained a passive avoidance memory. The behavioral assessments were conducted by a single trained investigator who was blinded to the treatment procedures during the testing phase. Statistical analyses were performed using one-way ANOVA with GraphPad Prism version 6.01.

### Molecular docking studies

The molecular docking studies were carried out using induced-fit docking (IFD) protocols implemented in the Schrödinger Small-Molecule Drug Discovery Suite (Schrödinger, LLC, New York, NY, United States) (Zou et al., 2014). The compounds constructed using the builder panel in Maestro were subjected to ligand preparation with LigPrep (Schrödinger Release 2023-1: LigPrep, Schrödinger) under the default conditions. The x*-*ray crystal structures of hAChE (PDB: 4EY7) and hBChE (PDB: 4TPK) were retrieved from the Protein Data Bank (Cheung et al., 2012; Brus et al., 2014). The proteins were prepared using the Protein Preparation Wizard tool, where hydrogen atoms were added first, followed by the assignment of all atom charges and atom types. Finally, energy minimization and refinement of the structures were performed to 0.3 Å of root mean-squared deviation (RMSD) by applying the OPLS3e force field. The receptor grid was generated by defining the centroid of the cocrystallized ligand as the box center, and the option “Dock ligands similar in size to Workspace ligand” was selected to automatically define the box size. A van der Waals (vdW) radius scaling factor of 1.00 and a partial charge cutoff of 0.25 were also used. The OPLS3e force field was applied throughout the grid generation process. The compounds prepared using LigPrep were docked onto hAChE and hBChE using the IFD protocol, which considers flexibility for both compounds and receptors. For hAChE, the residues D74, W86, Y124, Y133, S203, W286, F295, F297, Y337, F338, and H447 lining the binding sites of AChE were retained as flexible. The initial docking protocol was set to employ a 0.50 vdW radius scaling factor, and the resulting top-20 orientations of each compound were taken. An extra*-*precision algorithm was employed to redock the compounds with the low-energy refined structures generated by the Prime molecular mechanics/generalized Born surface area (MM/GBSA) method.

### MM/GBSA study

The ligand-residue free energies of the binding calculations were obtained using the MM/GBSA method in the Schrödinger suite. The equation used to calculate the binding energy is as follows:

$$\Delta G_{\text{bind}}=\Delta E_{\text{MM}}+\Delta G_{\text{solv}}+\Delta G_{\text{SA}},$$

where ΔE_MM_ is the difference in minimized energies given by

$$\Delta E_{\text{MM}}=E_{\left(\text{complex}\right)}\;‐\;E_{\left(\text{ligand}\right)}\;‐\;E_{\left(\text{receptor}\right)}$$

The difference in GBSA solvation energies between the complex and the sum of ligands and proteins is denoted by ΔGsolv. Furthermore, ΔGSA is the difference in the surface area energies between the complex and the sum of proteins and ligands. A script was used to calculate the average MM/GBSA binding energy, which also generates the Coulomb energy (Coulomb), covalent binding energy (Covalent), hydrogen-bonding energy (H-bond), lipophilic energy (Lipo), generalized Born electrostatic solvation energy (Solv_GB), and vdW energy.

### SwissADME-based prediction of drug-likeness and ADMET parameters

The selected compounds were comprehensively evaluated for their physicochemical properties, pharmacokinetic profiles, drug-likeness parameters, and medicinal chemistry characteristics through the SwissADME computational platform developed and maintained by the Molecular Modeling Group at the Swiss Institute of Bioinformatics (Daina et al., 2017). This web-accessible resource integrates numerous validated predictive algorithms to generate detailed absorption, distribution, metabolism, and excretion (ADME) profiles for the candidate molecules. The topological polar surface area (TPSA) was assessed by the fragment-based methodology established by Ertl et al. (2000) that allows efficient and precise estimates of the molecular polar surface area, which is a parameter widely utilized in predicting the transport characteristics of drug candidates. The lipophilicity assessment incorporated multiple predictive models for the octanol–water partition coefficient (log P_o/w_) by combining physics-driven approaches such as iLOGP, which reduces the overfitting tendency, with data-centered models trained on extensive experimental measurements. To enhance the reliability of prediction, a consensus log P value was calculated by integrating the outputs from multiple individual predictive models.

The toxicokinetic evaluation employed SwissADME’s integrated screening systems to identify structural motifs associated with pan-assay interference compounds (PAINS), which are known to generate false-positive signals in biological screening assays. Furthermore, Brenk filters were applied to identify potentially hazardous or chemically reactive functional groups that could compromise the compound safety or stability profile. The pharmacokinetic parameters, including substrate affinity for P-glycoprotein (P-gp) and inhibitory potential against cytochrome P450 (CYP450) enzyme isoforms, were predicted using support-vector-machine-based binary classification models embedded within the SwissADME framework. These predictive models were validated through 10-fold cross-validation protocols and independent test datasets and can be used to assess a compound’s probability of interacting with transport proteins and metabolic enzymes based on molecular descriptors and structural similarity to established substrates or inhibitors.

The skin permeability coefficient (log Kp) was calculated using the linear regression model established by Potts and Guy (1992), which correlates transdermal permeability with the molecular dimensions and lipophilicity parameters; more negative log Kp values are indicative of reduced capacity for passive diffusion across the epidermal layers. Additionally, the gastrointestinal (GI) absorption efficiencies and blood–brain barrier (BBB) penetration capabilities were evaluated using the BOILED-Egg classification model, which categorizes the compounds according to their predicted passive absorption and central nervous system access based on the apparent polarity (TPSA) and lipophilicity (WLOGP) values (Daina and Zoete, 2016). Within this visualization framework, compounds predicted to exhibit high GI absorption are represented in the “white” region, while those likely to traverse the BBB appear in the “yellow” (yolk) region. The model further indicates the P-gp substrate potential through distinct color-coded markers.

## Results

### ChE inhibitory effects of the phenolic compounds

As shown in Table 1, all compounds from various chemical groups were tested against 10 mM of the ChEs, of which 12 compounds were able to inhibit AChE, while 8 compounds achieved BChE inhibition over 50%. Among these, gallic acid, rosmarinic acid, 3-hydroxytyrosol, *trans*-resveratrol, EGCG, quercetin, and fisetin possessed the ability to inhibit both ChEs. The most active inhibitor against AChE and BChE was found to be quercetin (IC_50_: 1.22 ± 0.79 mM and 2.51 ± 0.04 mM, respectively). In our screening, the isoflavone (e.g., daidzein, genistein, and glycitein) and methoxyflavone (e.g., tricin, tangeretin, and diosmetin) derivatives as well as flavone O- and C-glycosides (e.g., isoquercitrin, orientin, and vitexin) were either completely inactive or exerted low inhibition capacities against both ChEs. Amentoflavone, myricetin, ellagic acid, and luteolin showed no inhibition toward the ChEs. However, caffeic acid (IC_50_: 3.51 ± 0.62 mM), apigenin (IC_50_: 3.52 ± 0.08 mM), and taxifolin (IC_50_: 7.18 ± 2.05 mM) were able to inhibit only AChE. Although several of the phenolic compounds were found to be promising ChE inhibitors, it is obvious that their inhibitory effects are lower than those of the reference drug galantamine.

### Antiamnesic activities of the phenolic compounds

To determine the antiamnesic activities of the compounds, we selected oleuropein, rosmarinic acid, gallic acid, EGCG, and 3-hydroxytyrosol for their dual ChE inhibition owing to their adequate quantities and conducted further evaluations using the passive avoidance test in mice as the prominent *in vivo* experimental model for memory deficit (Hornedo-Ortega et al., 2018). Here again, the animals were divided into eight groups (n = 8 per group) as follows: A: 0.9% NaCl (sham), B: scopolamine (control), C: oleuropein, D: 3-hydroxytyrosol, E: gallic acid, F: rosmarinic acid, G: EGCG, and H: galantamine (reference). During the acquisition period, the mice were first placed in the light compartment, followed by opening of the guillotine door 10 s later, and measurement of their transition times to the dark compartment using a digital timer (Table 2). After entering the dark compartment, the guillotine door was closed, and the mouse was retained on its feet for 3 s. Then, an electric current of 0.1 mA/10 g of body weight was applied throughout. Immediately after the learning period, only 0.9% NaCl was given to group A (sham) while groups B–G were administered the respective drugs through the *i.p.* route; to the animals in the H group, the reference drug galantamine was applied via *s.c.* injection at a dose of 10 mg/kg (Jang et al., 2008). After 30 min, scopolamine (1 mg/kg) was dissolved in 0.9% NaCl and administered by *i.p.* injection to groups B–G to induce amnesia. Twenty-four hours after the application, the mice were placed back in the light compartment, and their latency times were measured after opening the guillotine door. It was concluded that the mice that did not enter the dark compartment within 9 min (cutoff time) remembered the passive avoidance action. Our findings indicate that the aforementioned five phenolic compounds are effective in mice, with 3-hydroxytyrosol displaying the strongest antiamnesic effect.

**TABLE 2:** Doses administered to the experimental groups, weight averages, transition times to the dark compartment on the first and second days [mean (min) ± SEM], and statistical analysis results for the passive avoidance test.

Experimental groups		Dose (mg/kg)	Mean weight of mice (g)	Mean latency time (min) ± SEM^a^ (1st day)	Mean latency time (min) ± SEM^a^ (2nd day)
A	Sham		37	0.25 ± 0.04	3.52 ± 0.74
B	Control (scopolamine)	1	37	0.38 ± 0.04	0.91 ± 0.14
C	Oleuropein	20	40	0.39 ± 0.07	7.68 ± 0.55***
D	3-Hydroxytyrosol	20	37	0.44 ± 0.03	8.39 ± 0.31****
E	Gallic acid	20	39	0.29 ± 0.03	6.76 ± 1.11***
F	Rosmarinic acid	20	39	0.48 ± 0.11	6.02 ± 1.16**
G	EGCG	20	39	0.38 ± 0.04	4.01 ± 0.77^c^
H	Galantamine^b^	10	40	0.35 ± 0.08	6.42 ± 1.06***

^a^ Standard error of the mean.

^b^ Reference.

^c^ Not statistically significant: *p* > 0.05, ** Statistically significant compared to control group at *p* < 0.01, ***Statistically significant compared to control group at *p* < 0.001, **** Statistically significant compared to control group at *p* < 0.0001.

### Findings of the molecular docking studies with AChE

**FIGURE 1:**
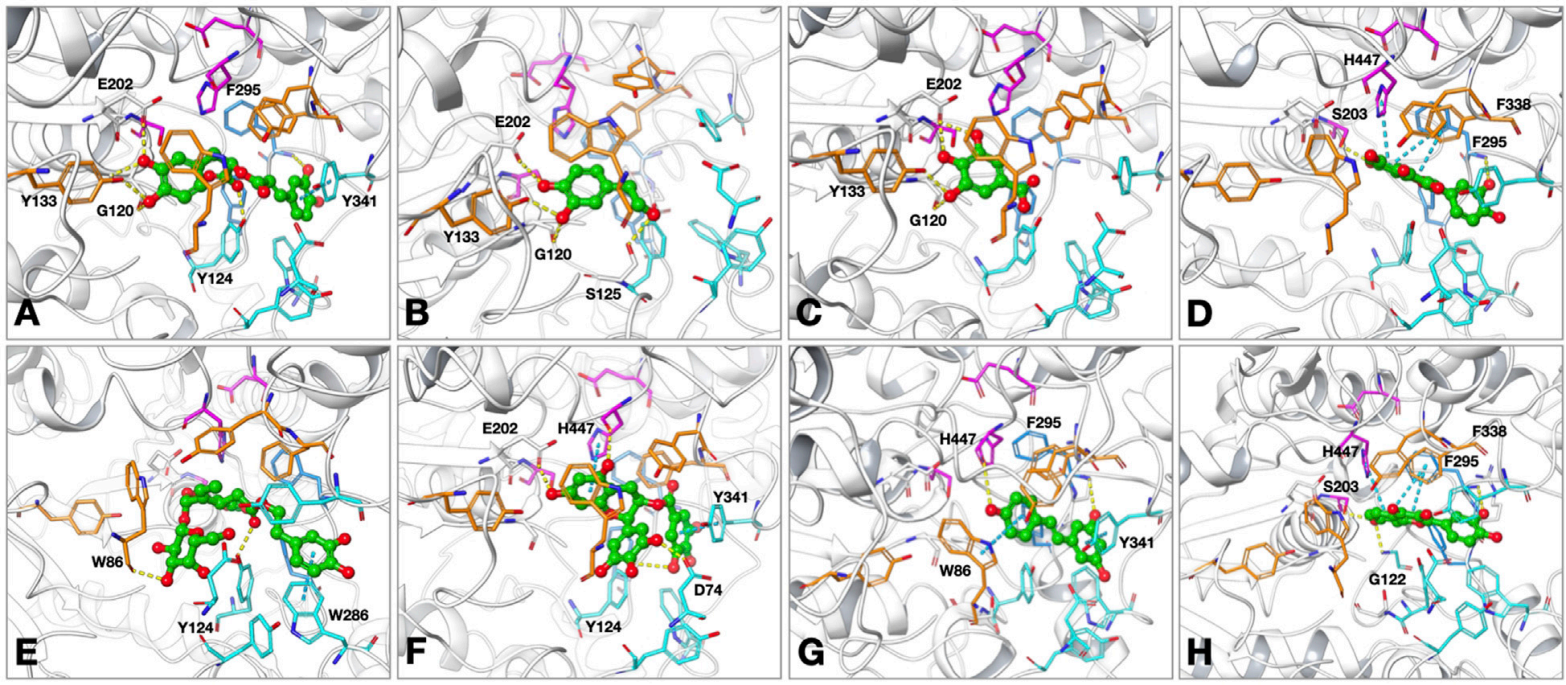
Proposed binding modes of **(A)** rosmarinic acid; **(B)** 3-hydroxytyrosol; **(C)** gallic acid; **(D)** quercetin; **(E)** oleuropein; **(F)** epigallocatechin gallate (EGCG); **(G)** resveratrol; **(H)** fisetin at the active site of human acetylcholinesterase (hAChE; PDB: 4EY7). The compounds are presented as green ball and stick models. The residues are colored as follows: catalytic triad: magenta, oxyanion hole: orange, acyl binding site: blue, peripheral anionic site: cyan. The hydrogen bonds and π–π interactions are shown by yellow and cyan dashed lines, respectively.

The molecular interactions of the selected compounds that displayed inhibitory effects against AChE were determined through the residues at its active site using the Glide module in Schrödinger suite. As seen from the results, the compounds reached the catalytic triad subunit of the enzyme active site and interacted mainly with the oxyanion region comprising residues (Figure 1). Relevantly, rosmarinic acid localized to the active site of AChE by occupying the oxyanion and PAS sites. The presence of hydrogen bonds was observed between the hydroxyl groups on the phenyl ring close to the carboxylic acid group and the residues G120, Y133, and E202. The carboxylic acid group interacted with Y124, a member of the PAS site, via hydrogen bonding. The other dihydroxyphenyl ring in the compound structure formed a π–π interaction with Y341 belonging to the PAS region. The binding energy for rosmarinic acid was calculated as −11.26 kcal/mol. Next, 3-hydroxytyrosol localized to the enzyme active site by interacting with the oxyanion site residues; the hydroxyl group at the third position of the phenyl ring was hydrogen bonded with Y133 and G120, while the hydroxyl group at the fourth position was hydrogen bonded with E202 and the hydroxyl group attached to the ethyl group was hydrogen bonded with S125 at the entrance of the active site. The binding energy for 3-hydroxytyrosol was calculated as −6.48 kcal/mol. As gallic acid is a small molecule, it penetrated the enzyme active site deeply and was located near the residues forming the catalytic triad; the docked complex was stabilized by hydrogen bonds formed between the hydroxyl groups and the residues G120, Y133, and E202. The AChE binding energy for gallic acid was calculated as −5.92 kcal/mol. Quercetin is oriented to the catalytic site of the enzyme with the coumarin ring; because of this orientation, hydrogen bonding and π–π interaction occurred between the coumarin ring and the catalytic triad members S203 and H447, respectively. Other π–π interactions stabilizing quercetin at the active site occurred between the coumarin ring and F338 belonging to the oxyanion site. In addition, the hydroxyl group at the third position of the phenyl ring attached to the coumarin ring interacted with the side chain of F295 in the acyl binding site via hydrogen bonding. The AChE binding energy for quercetin was calculated as −12.24 kcal/mol. While occupying the oxyanion site, oleuropein also interacts with the residues of the PAS site; the hydroxyl group at the fourth position on the tetrahydropyran ring in the structure interacted with the oxyanion site member W86 side chain via hydrogen bonding. The other interactions detected at the PAS site were the hydrogen bond between the carbonyl group and Y124 as well as the π–π interaction between the dihydroxyphenyl ring and W286. The binding energy for oleuropein was calculated as −11.58 kcal/mol. EGCG formed hydrogen bonds with the hydroxyl groups of the benzopyran ring E202 and the side chain of the catalytic triad member H447, and a π–π interaction was observed between the benzopyran ring and H447. The hydroxyl groups of the trihydroxyphenyl ring directly linked to the benzopyran ring formed hydrogen bonds with the PAS site member D74, whereas the gallate moiety showed a π–π interaction with Y341. The AChE binding energy for EGCG was calculated as −12.95 kcal/mol. The conformation of resveratrol was stabilized through hydrogen bonding with the side chains of F295 and H447 as well as π–π interactions with W86 and Y341. The binding energy for resveratrol was calculated as −9.14 kcal/mol. The binding mode of fisetin in the hAChE active site indicated that it formed π–π contacts with both the catalytic triad member H447 and oxyanion hole residue F338 through its chromen-4-one ring. The hydroxyl group in the seventh position on the same ring formed hydrogen bonds with S203 in the catalytic triad and G122 in the PAS region, while the hydroxyl group of the phenyl ring interacted with F295 in the acyl binding region via hydrogen bonding. The binding energy of fisetin at the AChE active site was calculated as −11.33 kcal/mol.

In the crystal structure of human AChE complexed with donepezil (PDB: 4EY7), we identified several key interactions between the ligand and residues lining the active site gorge. Notably, aromatic residues such as W86, Y124, W286, Y337, F338, Y341, and H447 formed hydrophobic and π–π contacts to stabilize the ligand within the gorge. Among these, W86, W286, and Y341 had some of the largest contact surface areas, effectively anchoring the ligand. In addition, a hydrogen bond was formed between the backbone or side chain of F295 and a polar group on donepezil, reinforcing its orientation near the catalytic center. Polar and charged residues like D74 and R296 are also involved in polar or ionic interactions, further enhancing the binding specificity. The compounds analyzed in the molecular docking simulations exhibited similar key interactions with the active-site residues and reflected comparable binding modes to that of the cocrystallized ligand.

### Findings of the molecular docking studies with BChE

**FIGURE 2:**
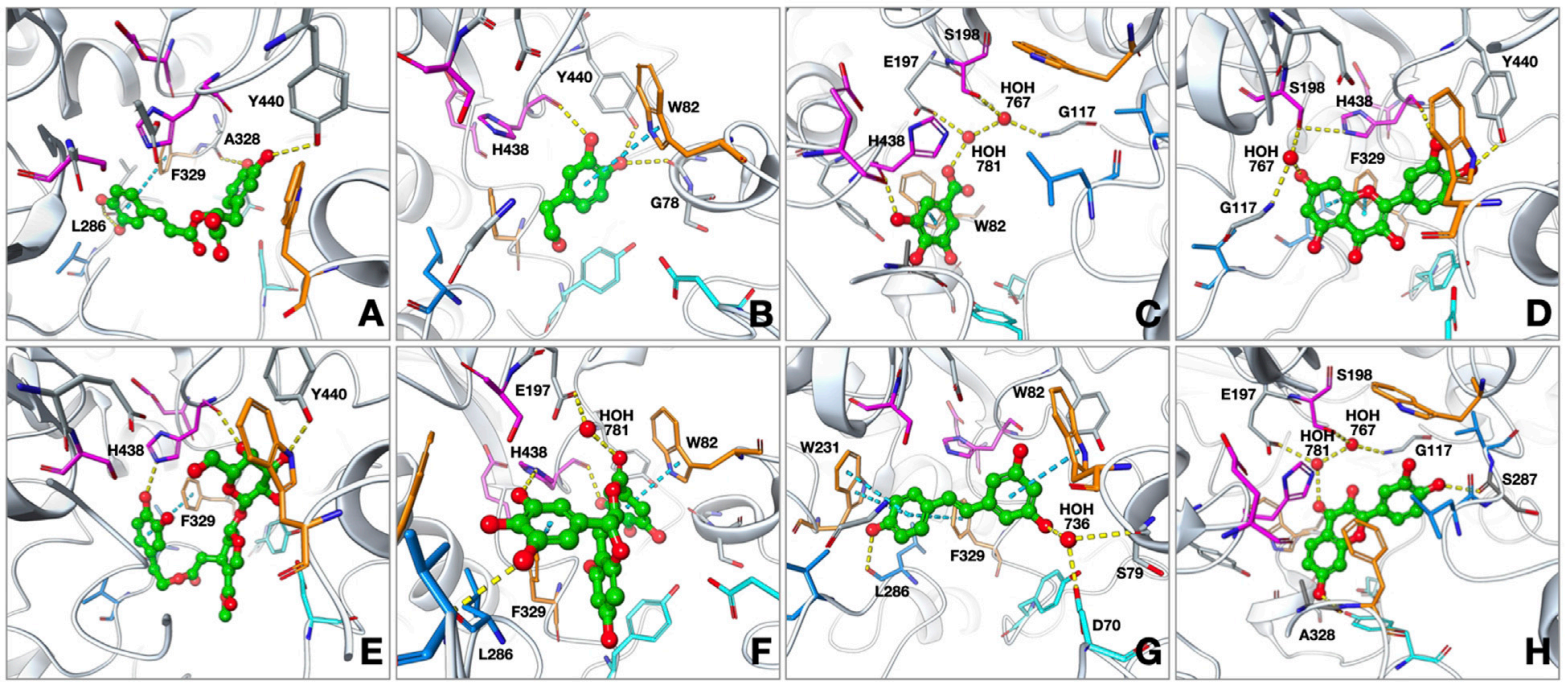
Proposed binding modes of **(A)** rosmarinic acid; **(B)** 3-hydroxytyrosol; **(C)** gallic acid; **(D)** quercetin; **(E)** oleuropein; **(F)** EGCG; **(G)** resveratrol; **(H)** fisetin at the active site of human butyrylcholinesterase (hBChE; PDB: 4TPK). The compounds are presented as green ball and stick models. The residues are colored as follows: catalytic triad: magenta, oxyanion hole: orange, acyl binding site: blue, peripheral anionic site: cyan. The hydrogen bonds and π–π interactions are shown by yellow and cyan dashed lines, respectively.

The interactions of the selected phenolic compounds that displayed inhibitory effects against BChE were investigated through the amino acids constituting the active site of the enzyme. Based on our outcomes from the molecular docking experiments, these compounds were patterned to reach the catalytic triad subunit of the active site and interacted predominantly with the residues belonging to the oxyanion hole (Figure 2). The conformation for rosmarinic acid at the BChE active site showed an interaction between the hydroxyl groups on the terminal phenyl ring and L286, a member of the acyl binding site, via hydrogen bonds; we also observed interactions between the hydroxyl groups on the other phenyl ring located close to the carboxylic acid group and A328 and Y440 via hydrogen bonds. In addition, the terminal phenyl ring showed a π–π interaction with F329. The BChE binding energy for rosmarinic acid was calculated as −10.39 kcal/mol. Next, 3-hydroxytyrosol was positioned in a region between the oxyanion site and the catalytic triad. The hydroxyl group at the third position of the phenyl ring formed a hydrogen bond with H438, a member of the catalytic triad, while the other hydroxyl group on the ring interacted with G78 and Y440 via hydrogen bonding. The ring interacted with W82, a member of the oxyanion site, via π–π interactions. The binding energy for 3-hydroxytyrosol was calculated as −6.14 kcal/mol. Gallic acid formed π–π interactions between its phenyl ring and W82 as well as a hydrogen bond with the hydroxyl group in the third position on the ring and H438. The docked complex was additionally stabilized by water-bridged hydrogen bonds with G117, E197, and S198 via its carboxylic acid group. The BChE binding energy for gallic acid was calculated as −6.03 kcal/mol. Quercetin formed a water-mediated hydrogen bond network between the hydroxyl group on the coumarin ring and G117 as well as its catalytic triad member S198. In addition to π–π interactions between the coumarin ring and F329 at the oxyanion site, the hydroxyl groups of the phenyl ring interacted with H438 and Y440 via hydrogen bonding. The BChE binding energy for quercetin was calculated as −8.60 kcal/mol. Oleuropein approached the catalytic triplet of the enzyme active site by forming direct hydrogen bonds between the hydroxyl groups on the tetrahydropyran ring and H438 and Y440. The compound also occupied the PAS site with its u-shaped binding mode and attached to the oxyanion site through a π–π interaction between the terminal dihydroxyphenyl ring and F329. The BChE binding energy for oleuropein was calculated as −11.41 kcal/mol. EGCG formed hydrogen bonds between L286 and H438, with the trihydroxyphenyl ring directly attached to the benzopyran ring, as well as π–π interaction with F329. The terminal phenyl ring formed a π–π interaction with W82 as well as a water-mediated hydrogen bond with E197. Another interaction stabilizing the enzyme–inhibitor complex is the hydrogen bond between the hydroxyl group attached to the terminal phenyl ring and the side chain of H438. The BChE binding energy for EGCG was calculated as −13.52 kcal/mol. Resveratrol localized to the BChE active site by interacting with the oxyanion and acyl binding sites; the hydroxyphenyl ring showed strong π–π interactions with amino acids W231 and F329 as well as formed hydrogen bonds with L286 via the hydroxyl group. Resveratrol formed π–π interactions with W82 through the dihydroxyphenyl ring as well as water-bridged hydrogen bonds with S79 and D70 in the PAS through the hydroxyl group. The binding energy for resveratrol was calculated as −7.01 kcal/mol. Fisetin was accommodated in the BChE active site primarily through hydrogen bonding; the carbonyl oxygen of the chromen-4-one ring interacted with E197 and the catalytic triad member S198 via a water-mediated hydrogen bond network, while the hydroxyl group in the seventh position on the same ring formed a hydrogen bond with A328. Additionally, the hydroxyl group at the fourth position on the phenyl ring, which is attached to chromen-4-one, was anchored at the acyl binding site to form a hydrogen bond with S287. The binding energy for the fisetin–BChE complex was calculated as −8.18 kcal/mol. The cocrystallized ligand (3F9, PDB: 4TPK) was lodged deep within the active site gorge through π–π and π–cation interactions with F329, W231, and Y332; hydrogen bonds were also formed with D70 and E197. The interactions identified with the coligand were also observed for the tested compounds, along with additional contacts that further stabilized the binding.

To further validate the docking results, MM/GBSA binding free energy calculations were performed for all docked complexes using Prime from the Schrödinger suite. The calculated ΔG_bind_ values generally supported the docking-derived rankings. For AChE, donepezil (cocrystallized ligand, PDB: 4EY7) displayed the most favorable binding free energy of −103.49 kcal/mol. Among the phenolics, EGCG (−95.42 kcal/mol), fisetin (−59.01 kcal/mol), and quercetin (−56.49 kcal/mol) exhibited highly favorable binding energies, whereas gallic acid (−3.50 kcal/mol) and 3-hydroxytyrosol (−17.96 kcal/mol) showed comparatively weaker stabilization (Table 3). For BChE, the cocrystallized ligand 3FP (PDB: 4TPK) had a ΔG_bind_ value of −86.11 kcal/mol, similar to oleuropein (−83.89 kcal/mol) and EGCG (−75.68 kcal/mol); in contrast, gallic acid (−2.51 kcal/mol) and 3-hydroxytyrosol (−27.68 kcal/mol) exhibited notably weaker binding energies (Table 4). These data validate the docking results and quantitatively position the phenolic compounds relative to donepezil in AChE and with respect to the reference ligand in BChE.

**TABLE 3:** Docking scores and molecular mechanics/generalized Born surface area (MM/GBSA) binding free energy components of the docked compounds and donepezil against AChE (PDB: 4EY7).

Compounds	Docking score (kcal/mol)	MM/GBSA dG bind	MM/GBSA dG bind coulomb	MM/GBSA dG bind covalent	MM/GBSA dG bind hbond	MM/GBSA dG bind lipo	MM/GBSA dG bind solv_GB	MM/GBSA dG bind vdW
Rosmarinic acid	−11.26	−22.63	73.23	11.66	−2.52	−30.33	−23.58	−48.43
3-Hydroxytyrosol	−6.48	−17.96	−20.88	7.96	−1.50	−14.74	33.06	−21.35
Gallic acid	−5.92	−3.50	65.51	4.32	−1.91	−9.31	−40.25	−21.39
Quercetin	−12.24	−56.49	−26.60	2.55	−1.22	−23.69	30.35	−35.69
Oleuropein	−11.58	−42.99	−36.46	15.99	−1.53	−53.64	68.99	−33.18
EGCG	−12.95	−41.60	−45.13	26.14	−2.63	−37.10	73.13	−52.44
Resveratrol	−9.14	−32.81	−12.32	1.34	−0.49	−26.36	41.23	−32.06
Fisetin	−11.33	−59.01	−28.26	2.46	−1.22	−23.73	28.30	−34.38
Donepezil	−19.21	−103.49	−93.66	7.14	−0.72	−52.30	102.86	−57.83

**TABLE 4:** Docking scores and MM/GBSA binding free energy components of the docked compounds and the cocrystallized ligand (3FP) against BChE (PDB: 4TPK).

Compounds	Docking score (kcal/mol)	MM/GBSA dG bind	MM/GBSA dG bind coulomb	MM/GBSA dG bind covalent	MM/GBSA dG bind hbond	MM/GBSA dG bind lipo	MM/GBSA dG bind solv_GB	MM/GBSA dG bind vdW
Rosmarinic acid	−10.39	−33.42	48.43	0.02	−1.86	−23.92	−13.88	−41.29
3-Hydroxytyrosol	−6.14	−27.68	−21.82	4.12	−1.07	−16.12	26.64	−19.17
Gallic acid	−6.03	−2.51	31.64	1.31	−1.56	−8.86	−4.60	−18.51
Quercetin	−8.60	−27.63	−23.94	4.03	−0.90	−15.52	42.18	−32.56
Oleuropein	−11.41	−83.89	−35.89	8.32	−1.42	−43.65	47.62	−57.82
EGCG	−13.52	−75.68	−55.75	4.92	−1.75	−29.80	59.70	−49.44
Resveratrol	−7.01	−29.05	−23.35	5.33	−0.57	−20.86	39.86	−28.03
Fisetin	−8.18	−32.50	−18.46	1.99	−0.80	−16.80	36.49	−33.50
3FP	−12.49	−86.11	−51.63	2.93	−0.03	−50.02	67.01	−53.18

### Computational ADME, pharmacokinetic, and toxicokinetic predictions

**FIGURE 3:**
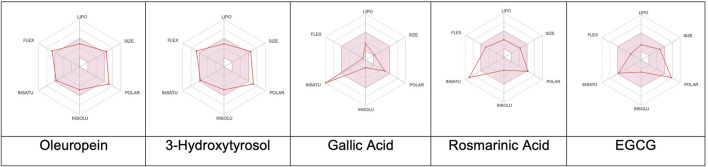
Colored zones showing the suitable physicochemical spaces for oral bioavailability of the corresponding compounds. LIPO (lipophilicity): −0.7 < XLOGP3 < 5; SIZE: 150 g/mol < molecular weight < 500 g/mol; POLAR (polarity): 20 Å^2^ < topological polar surface area < 130 Å^2^; INSOLU (insolubility): −6 < log S (ESOL) < 0; INSATU (insaturation): 0.25 < Csp^3^ fraction < 1; FLEX (flexibility): 0 < number of rotatable bonds < 9.

**FIGURE 4:**
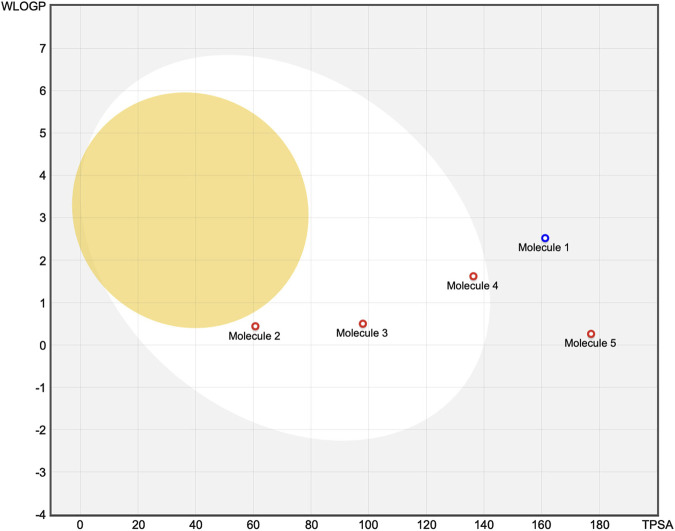
Molecules located in the BOILED-Egg yolk area (orange) are predicted to passively permeate through the blood–brain barrier. Molecules located in the white area are predicted to be passively absorbed by the gastrointestinal (GI) tract. Molecules located in the light-blue background area are neither absorbed by the GI tract nor cross the blood–brain barrier. The red dots are molecules predicted to not be effluxed from the central nervous system by P-glycoprotein. Molecule 1: oleuropein, Molecule 2: 3-hydroxytyrosol, Molecule 3: gallic acid, Molecule 4: rosmarinic acid, Molecule 5: EGCG.

The predictions revealed notable differences in the pharmacokinetic profiles of the five phenolic compounds analyzed (Figures 3, 4). Oleuropein exhibited suboptimal drug-likeness mainly owing to its high molecular weight (540.52 g/mol) and elevated TPSA (161.21 Å^2^), both of which exceeded the thresholds associated with good oral bioavailability; although its consensus log P value (2.41) indicated moderate lipophilicity, oleuropein was predicted to have low GI absorption with limited brain penetration and to be a substrate of P-gp, suggesting restricted cellular uptake. From the perspective of a structure–activity relationship (SAR), the absorption differences observed across the series can be rationalized by their physicochemical parameters. Oleuropein’s bulky glycosylated scaffold increases the polarity and hydrogen-bonding capacity, which together with its high molecular weight and flexibility reduces the passive permeability severely; in contrast, smaller phenolics like 3-hydroxytyrosol and gallic acid combine low TPSAs with limited rotatable bonds to enable efficient intestinal uptake. Rosmarinic acid represents an intermediate case, where favorable absorption is supported by its moderate lipophilicity despite the relatively high polarity. However, EGCG mirrors oleuropein in exhibiting excessive polarity and multiple hydrogen-bond donors, which correlate with its poor predicted absorption.

The SAR-based interpretations are aligned with the findings of the BOILED-Egg classification, where highly polar compounds cluster outside the white region of high GI absorption. In contrast, 3-hydroxytyrosol displayed favorable characteristics with a low molecular weight (140.14 g/mol), optimal TPSA (60.69 Å^2^), and balanced hydrophilic–lipophilic properties (log P: 0.60); it was predicted to be efficiently absorbed in the GI tract without crossing the BBB or interacting with P-gp. However, some potential CYP3A4 inhibition is suggested, indicating a possible risk of metabolic interactions. As the most hydrophilic compound in the series (log P: 0.21; TPSA: 97.99 Å^2^), gallic acid also shows high predicted GI absorption and no BBB permeability; similar to 3-hydroxytyrosol, it was not identified as a P-gp substrate but was predicted to inhibit CYP3A4. Rosmarinic acid presented intermediate properties with a molecular weight of 345.30 g/mol, TPSA close to the upper permeability limit (136.32 Å^2^), and moderate lipophilicity (log P: 1.17); despite its size and polarity, it was predicted to be well absorbed via the GI tract but not cross the BBB. No interactions with P-gp or the major CYP enzymes were anticipated. Lastly, EGCG had the highest TPSA (177.14 Å^2^) and a molecular weight of 394.33 g/mol, alongside a nearly neutral lipophilicity profile (log P: –0.03); these features correlated with poor predicted GI absorption and no BBB penetration. EGCG was also not expected to act as a P-gp substrate or CYP inhibitor.

In terms of water solubility, all compounds displayed favorable profiles ranging from soluble to very soluble according to the log S (ESOL) model; 3-hydroxytyrosol and gallic acid demonstrated the highest solubility with ESOL values of −1.48 and −1.64, respectively, which makes them very soluble. Oleuropein showed the lowest water solubility with an ESOL value of −4.76, making it “moderately soluble” while still being within an acceptable range for pharmaceutical formulations. The skin permeability coefficient (log Kp) values were negative for all compounds and ranged from −6.71 cm/s for 3-hydroxytyrosol to −8.39 cm/s for EGCG, indicating limited passive permeation across the skin. This suggests that topical formulations of these compounds would likely remain localized at the application site without significant systemic absorption.

### Findings from *in silico* toxicity screening

SAR analyses were performed to evaluate the mutagenic effects of the selected phenolic compounds. In the PASS program, the probabilities are obtained as P_a_ (probability of being active) and P_i_ (probability of being inactive) values. When these values are related as “P_a_ > P_i_” with the difference between them being large, the probability for the biological activity in question increases. In the SwissADME target prediction program, the probability for the biological activity in question is given directly as a percentage value. The screening results for the compounds that were active in the ChE inhibition assay as well as passive avoidance test are shown in Tables 3, 4. These compounds exhibited high probabilities of acting as Aβ aggregation inhibitors, which is another treatment strategy for AD. Apolipoprotein A1 (APOA1) is a structural protein from the group of high-density lipoproteins (Table 3); although the relationship between APOA1 and AD is complex and multifaceted, emerging evidence suggests that APOA1 may play a role in modulating the pathogenesis of AD. Given its involvement in lipid metabolism, inflammation, and neuroprotection, APOA1 has been proposed as a potential therapeutic target for AD (Endres, 2021). Therefore, it is notable that the compounds tested in our *in silico* analysis exhibited high probabilities for enhancing APOA1 expression. The *in silico* estimations of the compounds indicated the probabilities of common toxic effects like tremors, genotoxicity, vascular toxicity, hematemesis, and color changes to urine (Table 4).

To evaluate the potential metabolic interactions and off-target liabilities, the selected phenolic compounds were analyzed for their predicted inhibitions of the CYP450 enzymes as well as presence of structural motifs flagged by PAINS and Brenk filters. These computational alerts provide early indicators of potential assay interference, promiscuous binding, or chemical reactivity, which could compromise lead optimization or result in misleading biological outcomes. Among the compounds analyzed, 3-hydroxytyrosol and gallic acid were predicted to inhibit CYP3A4, one of the most clinically relevant isoforms involved in drug metabolism. This suggests the potential for drug–drug interactions when coadministered with substrates of this enzyme. In contrast, rosmarinic acid, EGCG, and oleuropein were not predicted to inhibit any of the major CYP enzymes evaluated, indicating a potentially lower risk of CYP-mediated interactions.

Despite the favorable CYP profiles for some of the compounds, all five molecules triggered at least one PAINS alert that was most associated with catechol_A substructures. According to Baell and Holloway (2010), such motifs are frequently linked to nonspecific activities across multiple biological targets, thereby increasing the risk of false-positive results in high-throughput screening assays. These findings underscore the importance of experimental validations to distinguish true pharmacological effects from assay artifacts. Additionally, Brenk alerts identifying chemically reactive or metabolically unstable functional groups were detected in several compounds. Specifically, 3-hydroxytyrosol, gallic acid, and EGCG triggered the catechol alert, while rosmarinic acid exhibited multiple alerts for catechol, hydroquinone, and michael_acceptor_1, indicating greater potentials for chemical reactivity or toxicity. Collectively, while the CYP inhibition profiles suggest a low risk of metabolic interactions for most of the compounds, the presence of PAINS and Brenk alerts highlights potential challenges in lead development. These findings emphasize the need for structural optimization and biological confirmation to ensure selective activity while reducing false-positive outcomes in early-stage drug discovery.

## Discussion

Phenolic compounds with various desirable biological effects for human health are among the most common secondary metabolite groups found in medicinal plants. According to our results from the passive avoidance test in mice with scopolamine-induced amnesia, the statistically most effective phenolic compound was found to be 3-hydroxytyrosol (Table 2). The results from our *in vivo* experiments were also consistent with the results of the ChE inhibition assays. The potential biological activity estimates of the phenolic compounds screened in this study display notable consistency with outcome of the *in silico* biological activity prediction program (PASS) (Table 3). Similarly, the results of our *in silico* molecular docking experiments show that the compounds identified as active ChE inhibitors have strong interactions with the amino acid units at the active sites of both enzymes (Figures 1, 2). The cumulative neuroprotective activity results show that oleuropein, 3-hydroxytyrosol, rosmarinic acid, gallic acid, and EGCG are the most useful phenolic compounds among those examined herein. Consistently, several studies have been reported on the neuroprotective effects of quercetin on ChE inhibition, including the study published previously by our group (Khan et al., 2009; Adedara et al., 2017; Abdel-Diam et al., 2019; Orhan, 2021). Moreover, there are other available studies on ChE inhibition by rosmarinic acid (Gulcin et al., 2016; Chao et al., 2022). For example, in a study examining the inhibitory effects of various herbal phenolics against ChEs, the inhibitory effects of the compounds were reported to be in the order rosmarinic acid > caffeic acid > gallic acid = chlorogenic acid (Szwajgier, 2015), which are compatible to our present results. It has also been reported that the presence of the CH=CH-COOH group has a highly positive effect on anti-AChE activity compared to the CH_2_-CH_2_-COOH or COOH group (Szwajgier, 2013), which is also consistent with the structures of the active inhibitory phenolic compounds in our screening.

In another study, rosmarinic acid was reported to exhibit ChE inhibitory effects (Gulcin et al., 2016), similar to our current prediction; the K_*i*_ values for rosmarinic acid were 42.52 pM and 121.60 pM for AChE and BChE, respectively. In our previous study, rosmarinic acid isolated from *Perovskia atriplicifolia* Benth. was shown to inhibit BChE selectively with an IC_50_ value of 6.59 ± 0.37 μg/mL (Senol et al., 2017), which is compatible with our present findings. In addition to the *in vitro* experiments carried out herein, data on the positive effects of rosmarinic acid on memory loss have been reported in various *in vivo* experiments involving ethanol-induced or lipopolysaccharide-induced cognitive dysfunction (Ozarowski et al., 2013; Hasanein et al., 2016; Fachel et al., 2020). In a study by Ozarowski et al. (2013), subchronic administration of the leaf extract of *Rosmarinus officinalis* L. to scopolamine-induced rats at a *p.o.* dose of 200 mg/kg indicated improvements in their long-term memory as well as AChE inhibition in the rat brains, which was attributed to rosmarinic acid. Pertinently, Hasanein et al. (2016) reported that rosmarinic acid as the main compound in *Salvia officinalis* L. exerted positive effects on memory and learning in normal and diabetic rats induced with streptozocin at doses over 400 mg/kg; rosmarinic acid was also found to increase the activities of superoxide dismutase (SOD) and catalase (CAT) enzymes while inhibiting lipid peroxidation and hyperglycemia.

Our screening shows that 3-hydroxytyrosol as one of the olive polyphenols could inhibit ChEs; it has been reported to exert remarkable neuroprotective effects in the SH-SY5Y cell line (Omar et al., 2018). In fact, 3-hydroxytyrosol as well as caffeic and chlorogenic acids have been found to effectively inhibit BChE as well as prevent protein and lipid oxidation (Roche et al., 2009). According to our literature survey, the neuroprotective effects of 3-hydroxytyrosol have been studied using the passive avoidance test in mice in the current study for the first time. It is also worth mentioning that 3-hydroxytyrosol was shown to display neuroprotection, particularly toward AD, through various action mechanisms. For instance, it caused significant decreases in lactate dehydrogenase efflux in a dose-dependent manner in rat models with hypoxic brains following 7 d of treatment at daily doses of 5 and 10 mg/kg (González-Correa et al., 2008). The compound also showed neuroprotective effects by enhancing mitochondrial dysfunction (Zheng et al., 2015), decreasing the formation of Aβ plaques (Nardiello et al., 2018), inhibiting microglia-mediated neuroinflammation (Khan et al., 2009), blocking oxidative damage (Rizzo et al., 2017), and modulating the hippocampal neuronal caspase-dependent apoptotic pathway (Arunsundar et al., 2015).

Oleuropein is another olive polyphenol that has been reported to have neuroprotective effects through assorted mechanisms, such as prevention of tau fibrillization and neuroinflammation (Chandler et al., 2010; Romero-Márquez et al., 2023). An equal mixture of oleuropein aglycone and hydroxytyrosol was tested in a cellular model of AD via exposure of the Aβ_1–42_ oligomers to a human SH-SY5Y cell line (Leri et al., 2021). This mixture led to a significant time-dependent reduction in the level of p62, a protein involved in AD, due to autophagic degradation through the binding of the autophagy marker LC3. Mnafgui et al. (2021) reported that oleuropein had the ability to alleviate brain damage in male Wistar rats that had experienced ischemic stroke. The compound improved the oxidative status by elevating the levels of SOD, CAT, and glutathione peroxidase while notably diminishing the levels of plasma fibrinogen and cardiac dysfunctional enzymes and inhibiting angiotensin-converting enzyme. In another work, oleuropein administration at doses of 10 and 20 mg/kg (*p.o.*) was shown to reverse amnesia in adult male Wistar rats (Hosseini et al., 2019). Oleuropein also had memory-improving effects at doses of 10, 15, and 20 mg/kg (*p.o.*) in colchicine-induced cognitive dysfunction, as demonstrated through the Morris water maze test in adult male rats (Pourkhodadad et al., 2016). The previous and current data confirm the neuroprotective potential of oleuropein at the preclinical level.

Gallic acid was found to be active in both of the ChE inhibition assays along with the passive avoidance test herein; it was consistently reported to be effective in a similar test in male rats treated at 10 and 20 mg/kg doses, which led to memory enhancement measured via the brain oxidant status and malondialdehyde (MDA) level (Azadeh et al., 2019). The same research group also showed the neuroprotective effects of gallic acid at doses of 5, 10, and 20 mg/kg over 21 d in stress-induced memory deficit in BALB/c mice, which led to a decrease in the elevated MDA levels in the mice brains (Salehi et al., 2018). Gallic acid also exhibited beneficial effects in ischemic rats with brain damage by augmenting memory, as assessed by the passive avoidance test, as well as cell viability in the hippocampus and cortex (Sarkaki et al., 2014). Gallic acid administered at doses of 50, 100, and 200 mg/kg (*p.o.*) for 10 d also displayed enhancement of memory deficit and cerebral oxidative stress levels induced by 6-hydroxydopamine in male adult Wistar rats (Wu et al., 2022); gallic acid was also reported to inhibit AChE on the same compound (Mansouri et al., 2013), as per our current finding.

Gallocatechins like (-)-epigallocatechin, (-)-epicatechin, (-)-epicatechin-3-gallate, and EGCG are the main bioactive polyphenols in *Camellia sinensis* L. Among these, EGCG has been reported to display neuroprotection through various molecular mechanisms. It modulates the aggregation of Aβ oligomers, detaches the fibrils deprived of increasing toxic intermediates, possesses a metal-chelation effect, suppresses neuroinflammation, and prevents oxidative stress, among others (Youn et al., 2022). Consistent with our data, Roche et al. (2009) identified EGCG as a notable inhibitor of human-serum-albumin-bound BChE, in addition to 3-hydroxytyrosol, oleuropein, quercetin, caffeic acid, and chlorogenic acid. These compounds were also able to prevent lipid peroxidation. In another study (Okello and Mather, 2020), EGCG markedly inhibited both ChEs with IC_50_ values of 0.0148 μmol/mL and 0.0251 μmol/mL. EGCG was also tested for its antiamnesic activity on male Sprague–Dawley rats having scopolamine-induced amnesia (Kim et al., 2021); these rats were administered EGCG by *i.p.* injection at a dose of 5 mg/kg/d for 10 d and showed improved memory deficit caused by scopolamine, similar to our current findings. In the same study, EGCG was shown to decrease AChE activities in the hippocampi of the rat brains. Furthermore, EGCG administered at doses of 20 and 40 mg/kg/d for 7 weeks was reported to alleviate memory and learning in streptozocin-induced amnesic rats (Baluchnejadmojarad and Roghani, 2011). EGCG was also reported to cause a dose-dependent decline in Aβ_1–42_-induced memory dysfunction evaluated via the passive avoidance and water maze tests (Lee et al., 2009). Eventually, the results of several previous studies and our current study indicate that EGCG is a remarkable herbal neuroprotective agent.

There are very few reported studies on the ChE inhibitory effects of fisetin. In an earlier study by Katalinić et al. (2010), several selected flavonoid derivatives comprising galangin, kaempferol, quercetin, myricetin, fisetin, apigenin, luteolin, and rutin were screened for inhibition of human BChE; of these, the most potent inhibitor was revealed to be galangin. Many studies have reported that the remarkable ChE inhibitory effects of flavonoids depend on the numbers and locations of the hydroxyl groups in their structures. Consistent with the results of our docking experiments, the inhibitory compounds were shown to bind to the active site of BChE by interacting with various hydrogen bonds and π–π stackings. In a recent study by Shi et al. (2024), a combination of fisetin and galantamine was shown to augment the binding affinity between AChE and galantamine, which led to a mixed type of inhibition through a synergistic action. Since fisetin was available only in a small amount in our laboratory, we were unable to evaluate it in the passive avoidance test. Nevertheless, fisetin has been disclosed to exhibit neuroprotection in an Aβ_1–42_-induced rat model of AD through the passive avoidance and Morris water maze tests (Wang et al., 2023); here, the compound was administered to the rats by the *i.g.* route at doses of 100, 50, and 25 mg/kg, and the hippocampal SOD and CAT levels were significantly increased in the fisetin-treated rats. Fisetin has also been shown to enhance choline acetyltransferase and diminish the activity of AChE, which are in agreement with our current findings.

SARs are undoubtedly important for evaluating the ChE inhibitory effects of phenolics. It was reported that the presence of a hydroxyl group, especially in the A ring of the flavonoid, as well as the double bond between C2 and C3 increased the affinity of the enzyme (hydrogen bonds) and enhanced the AChE inhibitory properties of flavonoids (Smyrska-Wieleba and Mroczek, 2023). The fact that the presence of a methoxy group negatively influences the activity of a flavonoid may also explain the null or low ChE inhibitory activities of the methoxyflavones tested herein (Lee et al., 2009). Consistent with our findings, apigenin has been described as an AChE inhibitor (Xie et al., 2014; Islam et al., 2021). In fact, the presence of the double bond between C2 and C3 in the structure of apigenin was concluded to decrease the affinity for AChE. Taxifolin (dihydroquercetin) has been consistently reported by several researchers to inhibit AChE effectively (Gocer et al., 2016; Kuppusamy et al., 2017; Lee et al., 2018).

We conducted *in silico* toxicity analyses on seven compounds that showed dual inhibition out of the 37 phenolics in the present work. The probabilities for acetylcholine neuromuscular blocking, cystathionine-β-synthase (CBS) inhibition, Aβ aggregation inhibition, and APOA1 expression enhancement were observed to be high for most of these seven compounds. CBS is a key enzyme involved in the metabolism of methionine and cysteine that plays critical roles in maintaining the sulfur amino acid balance, regulating homocysteine levels in the body, and contributing to the production of hydrogen sulfide (H_2_S) in the brain. Dysregulation of CBS activity has significant implications for health problems, such as intellectual disability, vascular thrombosis, skeletal abnormalities, and eye abnormalities. Relevant to our *in silico* findings, EGCG has been reported to strongly inhibit CBS (Zuhra et al., 2022), whereas there are no available data for the remaining CBS inhibitory phenolics tested herein. However, our *in silico* evaluations provide a strong hint that the other tested phenolic compounds may also have CBS inhibitory effects in the real experimental environment. Moreover, findings from previous studies show the Aβ aggregation inhibitory or anti-amyloidogenic activities of some of these compounds, such as resveratrol (Zuhra et al., 2022), fisetin (Aarabi and Mirhashemi, 2017), rosmarinic acid (Taguchi et al., 2017), EGCG (Fernandes et al., 2021), gallic acid (Liu et al., 2013), and 3-hydroxytyrosol (Chaari, 2020), which are in line with our *in silico* results. Considering their enhancement effects on APOA1, only one relevant study was available for EGCG, which selectively improved the mobilities of specific backbone and side-chain sites of APOA1 fibrils (Townsend et al., 2018). The *in silico* toxic or adverse effects of the dual inhibitory phenolic compounds are presented for the first time in the present study, which should be taken into consideration. Undoubtedly, the real toxicities of these phenolics may be different from our estimates; moreover, only limited amounts of data points are used to produce these predictions, whose precisions may change with more accessible data.

The optimal oral bioavailability for a drug candidate is influenced by a set of interrelated physicochemical parameters that define its favorable chemical space; these include lipophilicity (XLOGP3 between −0.7 and +5.0), molecular weight (150–500 g/mol), TPSA (20–130 Å^2^), aqueous solubility (log S of 6 to 0), molecular saturation (csp^3^ fraction of 0.25–1), and limited molecular flexibility (0–9 rotatable bonds). Together, these parameters support a balance between solubility, permeability, metabolic stability, and binding efficiency, thereby optimizing the likelihood of oral bioavailability (Lipinski et al., 2012). The SARs across the series further illustrate how deviations from the optimal physicochemical window translate to distinct pharmacokinetic profiles. Oleuropein violates multiple criteria simultaneously, namely molecular weight, polar surface area, and hydrogen-bonding capacity, resulting in poor drug-likeness and limited absorption. EGCG shares a similar liability owing to excessive polarity and hydrogen-bond donors. Conversely, 3-hydroxytyrosol and gallic acid fit well within the recommended absorption window, highlighting how small sizes and balanced polarity strongly favor bioavailability. Rosmarinic acid demonstrates a borderline profile; although its TPSA approaches the upper limit, the moderate lipophilicity compensates to maintain adequate absorption. Collectively, these trends emphasize the central roles of polarity, flexibility, and molecular size in shaping the absorption potential and reinforce the predictive power of *in silico* ADME models when interpreted in the SAR framework. While the Lipinski’s Rule of Five remains a cornerstone for evaluating oral drug-likeness (molecular weight < 500 g/mol, MLOGP < 4.15, hydrogen bond acceptors ≤10, hydrogen bond donors ≤5), natural products often fall outside these bounds yet retain their bioactivities and therapeutic relevance (Grabowski et al., 2008).

The evaluations of the five selected phenolic compounds reveal various levels of compliance with the drug-likeness criteria (Figure 3). Oleuropein violates Lipinski’s rule in two respects by exceeding both the molecular weight and allowed number of hydrogen bond acceptors; it also fails the Ghose, Veber, Egan, and Muegge filters, primarily owing to its size and high polarity/TPSA. Consequently, a low oral bioavailability score (0.17) is assigned to oleuropein, indicating its limited drug-likeness and significant formulation challenges. In contrast, 3-hydroxytyrosol adheres to all of the Lipinski criteria. Despite its small molecular size violating the Ghose and Muegge rules, it achieves a moderate bioavailability score (0.55). However, the presence of a catechol moiety resulted in one PAINS alert (catechol_A) and one Brenk alert (catechol), indicating potential assay interference or reactivity. Gallic acid exhibited a nearly identical profile as that of 3-hydroxytyrosol, fully complying with Lipinski’s rule and receiving a bioavailability score of 0.56. Similar to 3-hydroxytyrosol, it triggered the same PAINS and Brenk alerts, reflecting similar structural concerns. Rosmarinic acid demonstrated broader compliance by passing the Lipinski, Ghose, Veber, and Muegge criteria and violating only Egan’s rule owing to the TPSA exceeding the optimal range slightly; despite these minor deviations, its bioavailability score was also 0.56. However, multiple structural alerts were identified, including catechol_A (PAINS) and catechol, hydroquinone, and michael_acceptor_1 (Brenk), suggesting a higher probability of chemical reactivity or toxicity. EGCG violated one Lipinski criterion (hydrogen bond donors > 5) and failed the Veber, Egan, and Muegge rules owing to its high TPSA and hydrogen-bonding capacity; despite these, it received a comparable bioavailability score (0.55). EGCG also exhibited the same PAINS and Brenk alerts (catechol-based) observed in the other polyphenolic compounds screened herein.

In terms of the synthetic accessibility model, 3-hydroxytyrosol (score: 1.00) and gallic acid (1.22) were identified as the most synthetically tractable molecules; rosmarinic acid (2.58) and EGCG (4.13) were moderately accessible, while oleuropein (5.43) was the most challenging to synthesize, which could potentially complicate its development and scalability. Together, these findings highlight a broader SAR principle; polar polyphenols with extended hydrogen-bonding networks tend to show limited passive permeabilities, while compact phenolic scaffolds with fewer rotatable bonds achieve higher absorption efficiencies. This pattern is particularly relevant for natural products, which often lie outside the classical drug-likeness filters but can still inspire rational optimization. From a medicinal chemistry perspective, strategies such as reducing the TPSA, masking the hydrogen-bond donors/acceptors, and constraining the molecular flexibility may enhance the oral bioavailability. In this manner, SAR-informed modifications can guide future efforts to improve the pharmacokinetic profiles of structurally complex phenolics like oleuropein without compromising their biological activities.

Our preclinical data, which includes the *in vitro*, *in silico*, and *in vivo* animal model results, serve as the scientific foundation for the pharmaceutical potentials of the active compounds investigated herein. In particular, the findings on 3-hydroxytyrosol are a crucial first step in its potential translation from a natural product to a therapeutic agent. By demonstrating its significant *in vivo* antiamnesic activity, our work provides compelling evidence that warrants further investigation into its use as a lead compound for a drug molecule or as a nutraceutical to support cognitive health. The next critical steps would therefore entail detailed toxicological assessments and human clinical trials to fully evaluate its safety and efficacy.

## Conclusion

The present study provides a comprehensive evaluation of the neuroprotective potentials of several plant phenolics based on a multifaceted approach integrating *in vitro*, *in vivo*, and *in silico* methodologies. The *in vitro* screening revealed that several compounds, including gallic acid, rosmarinic acid, 3-hydroxytyrosol, *trans*-resveratrol, EGCG, quercetin, and fisetin, exhibit significant inhibitory activities against both AChE and BChE, suggesting their potential to modulate cholinergic neurotransmission. Furthermore, the *in vivo* passive avoidance test demonstrated the antiamnesic effects of oleuropein, rosmarinic acid, gallic acid, EGCG, and particularly 3-hydroxytyrosol in scopolamine-induced amnesic mice, highlighting their ability to ameliorate memory deficits. The molecular docking studies further elucidated the interactions of these active compounds with the active sites of AChE and BChE, providing insights into their binding modes and potential action mechanisms. Notably, the *in silico* ADME, pharmacokinetic, and toxicokinetic predictions offer a preliminary assessment of their drug-likeness and safety profiles. Although some compounds show lower ChE inhibitory activities than the reference drug galantamine, the identification of 3-hydroxytyrosol as a novel and potent ChE inhibitor with significant *in vivo* antiamnesic activity is a noteworthy finding. The favorable ADME and pharmacokinetic profiles predicted for 3-hydroxytyrosol further support its potential as a hopeful drug candidate. Future research efforts should also prioritize detailed mechanistic studies to fully elucidate the neuroprotective mechanisms of 3-hydroxytyrosol as well as *in vivo* toxicological assessments and clinical trials to translate these promising findings into effective treatments for neurodegenerative diseases.

### Study limitations

The main limitations of the present study are as follows. Although this study acknowledges the ChE inhibition potentials of several phenolic compounds, the inhibitory effects were generally lower than that of the reference drug galantamine. The *in vivo* experiments were conducted on scopolamine-induced amnesic mice, which is a widely used model for studying memory impairment that may not fully replicate the complex pathology of AD. Therefore, we recognize that the lack of dual-observer scoring is a limitation. The lack of detailed pharmacokinetic information and optimal dosing schedules for each molecule should be considered a limitation and warrants further investigations combining behavioral and pharmacokinetic assessments. The *in silico* toxicity assessment provides valuable predictive insights. However, further experimental validations are required to confirm the safety profiles of the active compounds. While the docking results offer valuable insights into the probable binding sites, a definitive characterization of the inhibition mechanism requires further investigation. We believe that a detailed mechanistic study, including kinetic analyses using methods like the Lineweaver–Burk plots, is a logical and important next step. Such future research would provide a more in-depth understanding of the precise interactions between these phenolic compounds and their target enzymes, further solidifying their therapeutic potentials.

## Data Availability

The raw data supporting the conclusion of this article will be made available by the authors, without undue reservation.

## Abbreviations

Aβ, amyloid beta; AChE, acetylcholinesterase; AD, Alzheimer’s disease; APOA1, apolipoprotein A1; BBB, blood–brain barrier; BChE, butyrylcholinesterase; CAT, catalase; CBS, cystathionine-β-synthase; ChE, cholinesterase; DTNB, 5,5′-dithio-bis (2-nitrobenzoic) acid; EGCG, epigallocatechin gallate; GI, gastrointestinal; IC, inhibitory concentration; IFD, induced-fit docking; *i.g.*, intragastric; *i.p.*, intraperitoneal; MDA, malondialdehyde; NMDA, *N*-methyl-D-aspartate; OPLS, optimized potential for liquid simulation; P-gp, P-glycoprotein; PASS, prediction of activity spectra for substances; PDB, protein data bank; *p.o.*, *per os* (oral); RMSD, root mean-squared deviation; SAR, structure–activity relationship; *s.c.*, subcutaneous; SD, standard deviation; SOD, superoxide dismutase; TPSA, topological polar surface area; vdW, van der Waals.
